# Organ‐based clues for diagnosis of inborn errors of immunity: A practical guide for clinicians

**DOI:** 10.1002/iid3.833

**Published:** 2023-04-12

**Authors:** Fatemeh Mohammadi, Amirhossein Yadegar, Mahta Mardani, Aryan Ayati, Hassan Abolhassani, Nima Rezaei

**Affiliations:** ^1^ School of Medicine Tehran University of Medical Sciences Tehran Iran; ^2^ Universal Scientific Education and Research Network (USERN) Network of Immunity in Infection, Malignancy and Autoimmunity (NIIMA) Tehran Iran; ^3^ Research Center for Advanced Technologies in Cardiovascular Medicine, Tehran Heart Center Tehran University of Medical Science Tehran Iran; ^4^ Research Center for Immunodeficiencies, Pediatrics Center of Excellence, Children's Medical Center Tehran University of Medical Sciences Tehran Iran; ^5^ Primary Immunodeficiency Diseases Network (PIDNet) Tehran Iran; ^6^ Children's Medical Center Tehran Iran

**Keywords:** clinical manifestation, diagnosis, guideline, inborn errors of immunity, primary immunodeficiency

## Abstract

Inborn errors of immunity (IEI) comprise a group of about 490 genetic disorders that lead to aberrant functioning or the development of distinct immune system components. So far, a broad spectrum of IEI‐related manifestations has been noted in the literature. Due to overlapping signs and symptoms of IEI, physicians face challenges in appropriately diagnosing and managing affected individuals. The last decade has witnesses improving in the molecular diagnosis of IEI patients. As a result, it can be the mainstay of diagnostic algorithms, prognosis, and possibly therapeutic interventions in patients with IEI. Furthermore, reviewing IEI clinical complications demonstrates that the manifestations and severity of the symptoms depend on the involved gene that causes the disease and its penetrance. Although several diagnostic criteria have been used for IEI, not every patient can be explored in the same way. As a result of the failure to consider IEI diagnosis and the variety of diagnostic capabilities and laboratory facilities in different regions, undiagnosed patients are increasing. On the other hand, early diagnosis is an almost essential element in improving the quality of life in IEI patients. Since there is no appropriate guideline for IEI diagnosis in different organs, focusing on the clues in the patient's chief complaint and physical exams can help physicians narrow their differential diagnosis. This article aims to provide a practical guide for IEI diagnosis based on the involved organ. We hope to assist clinicians in keeping IEI diagnosis in mind and minimizing possible related complications due to delayed diagnosis.

## Graphical abstract

Several manifestations of inborn errors of immunity (IEI) can be misinterpreted as separate diseases and cause mismanagement. This poses physicians considerable challenges, not only regarding diagnosing but also in formulating a treatment plan. As a result of the failure to consider IEI diagnosis and the variety of diagnostic capabilities and laboratory facilities, undiagnosed patients are increasing. Stratifying these disorders through their involved organs and considering all aspects of the disease in each organ can lead to greater comprehension of IEI.

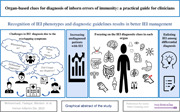

## 1 INTRODUCTION

Immune system defects constitute two major categories: primary (inherited) and secondary (acquired). Primary immunodeficiency, or inborn errors of immunity (IEI), predisposes individuals to an increased susceptibility to various combinations of recurrent infections, malignancy, autoimmunity, atopy, and lymphoproliferation.^1^ These inherited disorders influence both the humoral and cellular arms of the immune system. Up to now, about 490 IEI have been identified that affect different innate and adaptive immune system components.^2^ Many of them remain without a molecular diagnosis.^3^ Regarding the involved organ and the underlying pathophysiology, the clinical picture of IEI is highly variable. Clinical presentations can range from asymptomatic (identified by newborn screening or family genetic test) to severe immunological defects and sometimes manifestations in nonimmune organs.^4^, ^5^ Patients are often underdiagnosed because of overlapping signs and symptoms with a high degree of incomplete penetrance, pleiotropy, and epistasis due to different IEI genetic defects. Moreover, many cases with severe forms of the disorder may die due to complications before being assessed. So, the exact prevalence of IEI is still unclear. Since improper management significantly impacts the mortality rate of patients with IEI, the most critical step by a physician is a timely diagnosis of a susceptible patient, mainly in settings without IEI newborn screening. Suspicion toward immunodeficiency in the differential diagnosis results from awareness of the unspecific symptoms. Therefore, it is recommended that almost all general practitioners and specialists continue their recognition of IEI phenotypes and diagnostic guidelines.^1^ This article reviews common manifestations of IEI in different organs that guide the clinicians’ suspicion toward immunodeficiency (Table 1).

**Table 1:** Diagnostic clues for inborn errors of immunity (IEI) diagnosis.

Manifestation		Associated IEI
Respiratory	Pneumonia	XLA, CVID, CGD, SIgAD, CID, complement deficiency, PAD, X‐linked HIGM, WAS, NEMO deficiency, defects of antigen presentation, ADA deficiency, pulmonary alveolar proteinosis (due to CSF2RA and GATA2 deficiency), AT, NBS, PLAID, reduced serum Ig G2 level
	Organizing pneumonia or Bronchiolitis obliterans	WAS, DGS, XLP, CID, PAD, CVID
	Bronchiectasis	PAD, CF, CGD, Bloom syndrome, PGM3 deficiency, CHH, HIES, PCD, CVID, Ig GSD, defects of antigen presentation
	Bronchitis	CVID, XLA, IgG3 deficiency, IgG4 deficiency, SIgAD, defects of antigen presentation
	Atopy	dominant‐negative defects in CARD11, CVID, SIgAD
	Asthma	CHH, DOCK8 deficiency, SIgMD, CVID, SIgAD
	Chronic cough & pleurisy	CVID, SIgAD, XLA
Ear Nose Throat	Pharyngitis	HIES, SIgAD, SCID
	Laryngitis	HIES
	Mastoiditis	SIgAD, SAD, SCID, WAS, XLA, CVID, CGD
	Parotitis	SIgAD, isolated IgG3 subclass deficiency, CVID
	Deafness	PAD, Reticular Dysgenesis, ADA deficiency, SCID, Kabuki Syndrome, G6PC3 deficiency
	Sensorineural hearing loss	HIES with PGM3 mutations, DGS
	Otitis media	SIgAD, CVID, WAS, SCID, defects of complement components (C2, C3, H, I, P), THI, (Ig GSD), PCD, HIES, XLA, PAD, DGS
	Recurrent otitis externa	XLA, CGD
	Nasal polyposis	PCD, TAP deficiency, CF
	Rhinitis	SIgMD, CGD
	Sinusitis	complement deficiency, DGS, XLA, SIgAD, PCD, CF, HIES, CVID, SAD, SCID, AT
	Tonsillitis	x‐linked HIGM, CVID, APDS, SIgAD, SCID
	Adenotonsillar hypertrophy	AID and UNG deficiency, APDS, AT
Ophthalmology	Conjunctivitis	SIgAD, SIgMD, WAS, APECED, LAD type I, XLA
	Blepharokeratoconjunctivitis	CGD
	Blepharitis	HIES, SIgMD
	Recurrent staphylococcal hordeola	SIgMD
	Corneal scarring	CGD, WAS, APECED, XLA
	Keratitis	APECED, WAS, CGD
	Uveitis	CGD, Blau syndrome, MKD, CVID
	Macular edema	CGD
Hematology & Oncology	Autoimmune cytopenia	APDS, ALPS, CVID, SIgAD, STAT1 GOF, STAT3 GOF, DGS, IPEX, CTLA‐4 haploinsufficiency and deficiency of LRBA, WAS, dominant‐negative defects in CARD11, ICOS deficiency, PNP deficiency, SCID
	Pancytopenia	CHD, ALPS
	Anemia	CVID, SIgAD, ALPS, CHH, IPEX, WAS, CVID, XLP, SDS
	Neutropenia	CHH, GS2, WAS, DGS, Kostmann disease, Cohen Syndrome, WHIM syndrome, XLA, X‐linked CD40 ligand deficiency (the most common cause of HIGM), CVID
	Thrombocytopenia	IPEX, WAS, SDS, CVID, SIgAD, ALPS
	Idiopathic thrombocytopenic purpura (ITP)	CHH, CVID, DGS
	Eosinophilia	Omenn syndrome, HIES
	EBV‐associated lymphoma	Artemis syndrome, DGS, XLP, RASGRP1, CD70 and CD27, ITK, PRKCD, MST1, Coronin‐1A, CTPS1, CD137, MAGT1, CARMIL2 deficiencies
	Non‐Hodgkin lymphoma	CVID, NBS, CHH, AD‐HIES with STAT3 mutations
	Hodgkin's lymphoma	CD70 and CD27 deficiencies
	B cell lymphoma	DNA ligase IV deficiency, WAS, AD‐HIES with STAT3 mutations, RASGRP1 deficiency
	Burkitt lymphoma	AD‐HIES with *STAT3* mutations
	Disseminated anaplastic lymphoma	AD‐HIES with *STAT3* mutations
	mucosa‐associated lymphoid tissue lymphoma	DGS, CVID
	Myelodysplastic syndrome	WAS, GATA2 deficiency, Kostmann disease, SDS
	Acute lymphocytic leukemia	WAS, DGS, AT
	Acute myeloid leukemia	*GATA2* deficiency, AT, SDS
	Gastrointestinal cancer	Bloom syndrome, CVID, LS caused by mutations in any 1 of 4 genes (MLH1, MSH2, MSH6, and PMS2), DGS
	Skin cancer	Bloom syndrome, CHH, bone marrow failure
	Breast cancer	Bloom syndrome, AT, bone marrow failure
	Lung cancer	Bloom syndrome
	Ovarian cancer	Lynch syndrome caused by mutations in any 1 of 4 genes (MLH1, MSH2, MSH6, and PMS2)
	Endometrial cancer	Bloom syndrome, Lynch syndrome caused by mutations in any 1 of 4 genes (MLH1, MSH2, MSH6, and PMS2)
	HPV‐related papilloma cancer	EVER1, EVER2, MST1, RHOH, MAGT1, ITK deficiencies, WHIM syndrome
	Thyroid cancer	NBS, DGS, AT
	Osteosarcoma	Bloom syndrome
	Squamous cell carcinoma	HIES with DOCK8‐deficiency
	Basal cell carcinoma	CHH, HIES with DOCK8‐deficiency
Dermatology	Urticaria‐like rashes	APECED, PLAID, ADGRE2 Mutations, FCAS, MWS, NOMID, HIES
	Eczematous‐like rash	IPEX, WAS, Omenn syndrome, CHH, Netherton syndrome
	Papuloerythematous skin rash	Blau syndrome
	Psoriasis plaques	STAT1 GOF, CHH
	Hyperkeratotic plaques	PLS, CMC
	Hypo or hyperpigmented skin lesions	Bloom syndrome
	Sun‐sensitive skin lesions	Bloom syndrome, CGD
	Cafe.au.lait spots	WAS, NBS, ICF syndrome, PMS2, MSH2 deficiencies
	Livedo reticularis	ADA2 deficiency
	Granulomatous skin lesions	CHH, CVID, AT, HIES, MHC I deficiency
	Cutaneous viral infections	CARD11, LCK, NEMO, GATA2, STK4, DOCK8, STAT2 deficiencies, STAT1 GOF, Netherton syndrome, WHIM syndrome, WILD syndrome
	Acne, Cellulitis	CGD
	Boils/soft tissue abscesses	CGD, HIES, LAD
	Petechiae and Purpura	WAS
	Telangiectasia	AT, ataxia‐telangiectasia–like disorders, Bloom syndrome
	Vitiligo	STAT1 GOF, DGS
	Discoid lupus	female carriers of X‐linked CGD, complement deficiency
	Scleroderma	STAT3 GOF
	Atopic dermatitis	AD‐HIES (job's syndrome), IPEX, Netherton syndrome, PGM3 deficiency, DOCK8 deficiency, STAT3 GOF, Omenn syndrome, hypereosinophilia
	Alopecia	dominant‐negative defects in CARD11, STAT1 GOF, CVID, XLA, CMC, SCID, Omenn syndrome, hypereosinophilia
	Ichthyosis and trichorrhexis invaginatum	Netherton syndrome
	Nail problems	PLS, DKC, CMC
	Poor wound repair	LADs, *Rac2* mutation, *WDR1* deficiencies
Gastrointestinal	Gastroesophageal reflux	DGS, Bloom syndrome, STAT3‐HIES
	Esophageal dysmotility	STAT3‐HIES
	Esophageal atresia, Tracheoesophageal fistula, Laryngeal web	DGS
	Gastritis	CVID, APECED
	Hepatitis	APDS, APECED, IPEX, X‐linked HIGM, STAT1 GOF, STAT3 GOF
	Pyogenic liver abscess	PLS, CGD
	Cirrhosis	CVID, CD40 ligand deficiency
	Nodular regenerative hyperplasia (NRH)	CVID, APDS, XLA
	Abnormal liver biochemistries	CVID
	Sclerosing cholangitis	CD40 ligand deficiency
	Exocrine pancreatic insufficiency	SDS
	Hepatosplenomegaly	CHH, CGD, CVID, ALPS, hypereosinophilia, CHH
	Celiac disease	CVID, SIgAD
	Crohn's disease resemblance	CVID
	Inflammatory bowel disease	WAS, CGD, CVID, XLA, XIAP deficiency, NEMO deficiency
	Fungal infections	STAT3‐HIES
	Giardiasis	SIgAD, XLA
	Strongyloidiasis	SIgAD
	Spontaneous intestinal perforations	STAT3‐HIES
	Constipation	DGS, CGD
	Diarrhea	CGD, XLA, SCID, CHH, CVID, defects of antigen presentation
	Steatorrhea	SDS
	Intestinal malrotation, Hirschsprung disease, Imperforate anus, hernia	DGS
	Intestinal obstruction and vomiting	Immunodeficiency with multiple intestinal atresia
	Early‐onset enteric fistula	IL10, IL10RA, IL10RB deficiencies
	Hepatomegaly and veno‐occlusive disease	VODI syndrome
Endocrinology	Anterior pituitary disorders	CVID, Ig GSD, SPAD
	Hypoparathyroidism	DGS, 10p13‐p14DS, APECED, CMC, CHH, CVID
	Osteoporosis	CVID, HIES
	Growth hormone deficiency	SCID, CVID, SDS, HIES, CGD, DGS
	Hypogonadism	ICF syndrome, congenital agammaglobulinemia, Bloom syndrome, HIES, AT
	Thyroid dysfunction	STAT1 GOF, SIOD
	Thyroiditis	ADA deficiency, SIgAD, IPEX, WAS
	Hypo‐ or hyperthyroidism	CHH, DGS
	Adrenal insufficiency	MCM4, AIRE, CTLA4 gene mutation, NF‐kB dysfunction, DAVID syndrome, MIRAGE syndrome
	Diabetes mellitus	AT, CF, IEI with DNA repair impairments, Bloom syndrome, IPEX, WHIM syndrome, APS type 1, CTLA4 mutation, deficiency of LRBA, ADA deficiency, STAT1 GOF
Reproductive & urology	Premature ovarian failure	APECED, CVID
	Ovarian insufficiency	Kostmann disease (HAX1 gene mutation)
	Absent uterus	DGS
	Micropenis	CHARGE syndrome (CHD7 gene deficiency)
	Cryptorchidism, Hypospadias	DGS
	Absence of pubertal development	CHARGE syndrome (CHD7 gene deficiency)
	Pubertal delay	AT
	Premature thelarche	Kabuki Syndrome
	Early menopause	Bloom syndrome
	Infertility	Bloom syndrome, CF
	Spermatogenesis impairment	CHH
	Urinary tract malformation	Kabuki Syndrome, GATA 2 deficiency
Rheumatology	Piezogenic pedal papules	GATA2 deficiency
	Arthritis	X‐linked CD40L deficiency (the most common cause of HIGM), sIgAD, STAT3 GOF, Blau Syndrome, APDS, MHC I deficiency, ALPS, DGS, IPEX, WAS, XLA, ICOS deficiency, PNP deficiency, SCID
	Juvenile idiopathic arthritis	CHH, CVID
	Septic arthritis	XLA
	Systemic lupus erythematosus (SLE)	Complement deficiency (C1, C2, and C4), sIgAD, STAT1 GOF, CVID
	Vasculitis	MHC I deficiency, WAS, Blau syndrome, IPEX, HIES with DOCK8‐deficiency, CVID
	Sjogren's‐like disease	APECED, CVID
	Aphthous ulcers	X‐linked CD40L deficiency (the most common cause of HIGM)
	Oral ulcers	CGD, HIGM, CVID, SCID
Nephrology	Glomerulonephritis	APDS, complement deficiency, Complement Factor H Deficiency
	Atypical hemolytic‐uremic syndrome (HUS)	Complement Factor H Deficiency
	IgA nephropathy	WAS
	Progressive renal failure	SIOD
	Renal anomalies/agenesis, Hydronephrosis	DGS
	Granuloma of the kidneys	Blau syndrome
	Vesico‐renal‐genital anomaly	CHARGE syndrome, G6PC3 deficiency, 3MC syndrome
Cardiology	Pericarditis	APDS
	Aneurysms	STAT1 GOF, all subtypes of HIES
	Pseudoaneurysms	all subtypes of HIES
	Coronary artery tortuosity or dilation	STAT3‐mutated HIES
	Interrupted aortic arch	DGS
	Venous angeictasis	G6PC3 deficiency
	Angioedema	C1 inhibitor, factor XII deficiencies
	Thrombosis	all subtypes of HIES
	High cutoff level of peak systolic aortic pressure, Pulmonary arterial hypertension	CVID
	Superior vena cava syndrome	all subtypes of HIES
	Valvular regurgitation	CVID
	Murmur or cyanosis	DGS, SCN type 4, CHARGE syndrome, Kabuki Syndrome, MST1/STK4 deficiency
	Congenital heart disorder	DGS, SCN type 4, CHARGE syndrome, Kabuki Syndrome, G6PC3, MST1/STK4 deficiencies
	Congenital patent ductus venosus	all subtypes of HIES
	MISC after COVID‐19	IFNAR1 deficiency
Face	Facial abnormalities	DGS, ICF syndrome, 3MC syndrome type 2 (COLEC11 deficiency), Cohen syndrome (COH1 deficiency), ITCH deficiency, STAT5B deficiency, FILS syndrome, RNF168 deficiency, Kabuki Syndrome, Bloom syndrome, ICF, Cernunnos (XLF)
	Rough skin appearance with exaggerated pore size	HIES with STAT3 mutations
	Wide nostrils and prominent lips	HIES with PGM3 mutations
	Bird‐like face	NBS
Odontology	Retention of deciduous teeth	HIES
	Delay in the shedding of primary teeth	STAT3 deficiency
	Premature loss of teeth	CHD, LAD type I, PLS
	Dental enamel hypoplasia	APECED, DGS
	Gingivitis	neutrophil deficiency, CGD
	Stomatitis	HIGM, cyclic neutropenia, CGD
	Periodontitis	CHD, LAD type I, PLS
Psychology and intellectual disability	Neurocognitive disorders	DGS, Kostmann disease, HIES with PGM3 mutations, AT, syndromic CIDs
	Developmental delay	3MC syndrome, LAD type 2, Cohen syndrome, Kabuki Syndrome, Barth syndrome, P14 deficiency, ADAR1 deficiency, HIES with PGM3 mutations, PNP deficiency, MKD, DGS, NBS, ITCH deficiency
	Intellectual disability	DGS, b‐actin deficiency, HIES with PGM3 mutations, Kabuki Syndrome, Bloom syndrome, NBS, CHD, ICF syndrome
	ADHD, ASD, Anxiety, Mood disorders, Schizophrenia	DGS
Neurology	Meningitis	PAD, XLA, WAS, HIES with DOCK8‐deficiency, complement deficiency
	Intracranial calcifications	SPENCDI, ADAR1 deficiency, SAMHD1 deficiency, type 1 interferonopathies
	Cerebral atrophy	SPENCDI, ADAR1 deficiency, SAMHD1 deficiency, type 1 interferonopathies
	Leukodystrophy	SPENCDI, ADAR1 deficiency, SAMHD1 deficiency, type 1 interferonopathies
	Progressive multifocal leukoencephalopathy	PNP deficiency
	Encephalopathy	CVID
	Viral encephalitis	TLR3, UNC93B1, TRAF3, TRIF, TBK1 deficiencies
	Seizures	DGS, Kabuki Syndrome
	Neuropathy	CVID, CHD, Blau Syndrome, CHH
	Myelopathy	CVID
	Fibromyalgia	IgGSD
	Brain Aneurysms	STAT3‐mutated HIES
	Brain infarction	HIES with DOCK8‐deficiency, ADA2 deficiency
	CNS vasculitis	XLP
	Spontaneous CNS candidiasis	CARD9 deficiency
	Neurological defect	Kostmann disease
	Pyramidal signs	PNP deficiency
	Dysarthria	HIES with PGM3 mutations
	Myoclonus	HIES with PGM3 mutations
	Ataxia	MKD, HIES with PGM3 mutations, AT, AT‐like disease, PNP deficiency, RNF168 deficiency
	Increased serum level of a‐fetoprotein	AT, AT‐like disease, PNP deficiency, RNF168 deficiency
	Narcolepsy	CHH
	Microcephaly	NBS, DGS, DKC1, RNF168, RAD50, DNA ligase IV, Cernunnos (XLF), DNAPKcs deficiency
Orthopedy	Fractures following minor trauma	HIES
	Scoliosis	HIES, DGS, HIES with PGM3 mutations, Kabuki syndrome
	Degenerative spine disease	HIES
	Spinal deformity, Rib cage abnormalities	SDS
	Cervical and thoracic vertebral anomalies	DGS
	Craniosynostosis	HIES, DGS
	Dystrophy	CANDLE syndrome
	Chondrodysplasia	SDS
	Costochondral junction flaring	ADA deficiency
	Skeletal dysplasia	mutations in the RNF168, MCM4, ACTB, ACP5, STAT5B, FILS, SMARCAL1, RMPR, BLM genes
	Spondylo‐epiphyseal dysplasia	SIOD
	Hip dislocation	Kabuki Syndrome
	Limb dwarfism	mutations in the RNF168, MCM4, ACTB, ACP5, STAT5B, FILS, SMARCAL1, RMPR, BLM genes
	Short stature	FILS syndrome, mutations in the RNF168, MCM4, ACTB, ACP5, STAT5B, FILS, SMARCAL1, RMPR, BLM genes, SDS
	Bone and joint infections	CVID, XLA
	Fungal osteomyelitis of the skull base	MPO deficiency
	Neonatal sterile multifocal osteomyelitis	DIRA syndrome
	Osteomyelitis	CGD, XLA
	Camptodactyly	Blau syndrome
	Polydactyly, Club foot	DGS
	Joint hyperextensibility	GATA2 deficiency, HIES, Kabuki syndrome

Abbreviations: 10p13‐p14DS, chromosome 10p13‐p14 deletion syndrome; 3MC, Malpuech, Michels, Mingarelli, Carnevale syndrome; ADA, adenosine deaminase deficiency; ADHD, attention deficit with hyperactivity disorder; AID, activation‐induced cytidine deaminase; ALPS, autoimmune lymphoproliferative syndrome; APDS, activated phosphoinositide 3‐kinase δ syndrome; APECED, autoimmune polyendocrinopathy candidiasis ectodermal dystrophy; APS type 1, autoimmune polyglandular syndrome type 1; ASD, autism spectrum disorder; AT, ataxia‐telangiectasia; CANDLE, chronic atypical neutrophilic dermatosis with lipodystrophy and elevated temperature syndrome; CF, cystic fibrosis; CGD, chronic granulomatous disease; CHARGE, coloboma, heart defect, atresia of the choanae, retardation, genital, and ear abnormalities syndrome; CHH, cartilage‐hair hypoplasia; CHS, Chediak–Higashi syndrome; CMC, chronic mucocutaneous candidiasis; CNS, central nervous system; COVID‐19, coronavirus disease; CTLA‐4, cytotoxic T lymphocyte antigen 4; CVID, common variable immunodeficiency; DAVID syndrome, CVID accompanied with adrenocorticotropic hormone (ACTH) insufficiency; DGS, DiGeorge syndrome or 22q11.2 deletion syndrome; DIRA, deficiency of the interleukin 1 receptor antagonist syndrome; DKC, dyskeratosis congenita; DNAPKcs, DNA‐dependent protein kinase catalytic subunit deficiencies; FCAS, familial cold autoinflammatory syndrome; FILS, facial dysmorphism, immunodeficiency, livedo, and short stature syndrome; G6PC3, glucose‐6‐phosphatase enzyme deficiency; GOF, gain‐of‐function; GS2, griscelli syndrome type 2; HIES, hyper‐Ig E syndrome; HIGM, hyper IgM syndrome; HPS2, Hermansky‐Pudlak syndrome type 2; IFN‐gamma, interferon‐gamma; Ig GSD, IgG subclass deficiency; IL‐12, interleukin‐12; IPEX, immune dysregulation polyendocrinopathy enteropathy X‐linked; LAD, leukocyte adhesion deficiency; LRBA, lipopolysaccharide‐responsive beige‐like anchor; MALT, mucosa‐associated lymphoid tissue lymphoma; MHC, major histocompatibility complex I deficiency; MIRAGE, myelodysplasia, infection, restriction of growth, adrenal hypoplasia, genital phenotypes, and enteropathy syndrome; MISC, multisystem inflammatory syndrome; MKD, mevalonate kinase deficiency; MPO, myeloperoxidase deficiency; MWS, Muckle‐Wells syndrome; NBS, Nijmegen breakage syndrome; NF‐kB, nuclear factor kappa B; NOMID, neonatal‐onset multisystem inflammatory syndrome; PAD, primary antibody deficiency; PCD, primary ciliary dyskinesia; PGM3, phosphoglucomutase‐3 deficiency; PLCγ2, phospholipid‐specific phospholipase C‐γ2–associated antibody deficiency and immune dysregulation (PLAID); PLS, Papillon–Lefèvre syndrome; PNP, purine nucleoside phosphorylase deficiency; SAD, specific antibody deficiency; SCID, severe combined immunodeficiency; SD/THE, syndromic diarrhea/tricho‐hepato‐enteric syndrome; SDS, Shwachman–Diamond syndrome; SIgAD, selective immunoglobulin A deficiency; SIgMD, selective IgM deficiency; SIOD, schimke immuno‐osseous dysplasia; SPAD, specific anti‐polysaccharide antibody deficiency; TAP, transporter associated with antigen presentation deficiency; THI, transient hypogammaglobulinemia of infancy; UNG, uracil‐DNA glycosylase; VODI, veno‐occlusive disease with immunodeficiency syndrome; WAS, Wiskott‐Aldrich syndrome; WHIM, warts, hypogammaglobulinemia, infections, and myelokathexis syndrome; WILD, warts, depressed cell‐mediated immunity, primary lymphedema, and anogenital dysplasia syndrome; XIAP, X‐linked inhibitor of apoptosis deficiency; XLA, X‐linked agammaglobulinemia; also known as Bruton's agammaglobulinemia; XLP, X‐linked lymphoproliferative disease.

## 2 RESPIRATORY

### 2.1 Common

Atopy is a characteristic feature of selective immunoglobulin A deficiency (SIgAD), common variable immunodeficiency (CVID), and dominant‐negative defects in *CARD11*.^6^, ^7^, ^8^ Moreover, asthma can provide clues to the diagnosis of cartilage‐hair hypoplasia (CHH), CVID, SIgAD, *DOCK8* deficiency, and selective IgM deficiency (SIgMD).^9^, ^10^, ^11^, ^12^, ^13^ Chronic cough and pleurisy are noticed in CVID, X‐linked agammaglobulinemia (XLA), and SIgAD.^14^

Bronchiectasis is noticed in primary antibody deficiency (PAD), CVID, IgG subclass deficiency (Ig GSD), defects of antigen presentation, chronic granulomatous disease (CGD), cystic fibrosis (CF), primary ciliary dyskinesia (PCD), Bloom syndrome, Phosphoglucomutase‐3 (PGM3) deficiency, CHH, or Hyper‐IgE syndrome (HIES).^9^, ^11^, ^15^, ^16^, ^17^, ^18^, ^19^, ^20^, ^21^ Bronchitis is reported in CVID, XLA, SIgAD, defects of antigen presentation, IgG3 deficiency, and IgG4 deficiency.^20^, ^21^

Recurrent pneumonia is manifested in combined immunodeficiency disorders (CIDs), PAD, X‐linked hyper IgM syndrome (HIGM), Wiskott‐Aldrich syndrome (WAS), NEMO deficiency, CGD, XLA, CVID, defects of antigen presentation, complement deficiency, and SIgAD.^14^, ^17^, ^20^, ^21^, ^22^ Noninfectious pneumonia is seen in adenosine deaminase (ADA) deficiency and pulmonary alveolar proteinosis (due to *CSF2RA* and *GATA2* deficiency). Aspiration pneumonia is investigated in syndromic CID and concurrent neurological defects such as ataxia‐telangiectasia (AT), Nijmegen breakage syndrome (NBS), and other DNA repair defects.^20^ Interstitial pneumonia and bronchiolitis may help clinicians recognize phospholipid‐specific phospholipase C‐γ2 (PLCγ2)–associated antibody deficiency and immune dysregulation (PLAID).^23^ Lung abscesses are seen in CGD, leukocyte adhesion deficiency (LAD), HIES, and Chediak–Higashi syndrome (CHS).^13^, ^24^ Lower respiratory tract infections can indicate the probability of PCD and transient hypogammaglobulinemia of infancy (THI).^17^

Another manifestation of IEI is cancer, with non‐Hodgkin extra nodal B‐Cell lymphoma and metastasizes being the most common complications. Lymphoma could be a documented complication of SIgAD, CVID, XLA, WAS, DiGeorge syndrome (DGS), X‐linked lymphoproliferative disease (XLP), and AT.^13^, ^25^

### 2.2 Uncommon

Immune‐mediated interstitial lung disease (ILD) is reported in PAD, Ig GSD, SIgAD, cytotoxic T lymphocyte antigen 4 (CTLA‐4) haploinsufficiency and deficiency of lipopolysaccharide‐responsive beige‐like anchor (LRBA) or *STAT3* gain‐of‐function (GOF), CGD, and AT.^6^, ^13^, ^20^ Lymphoid interstitial pneumonitis is detected in patients with CVID.^26^ Granulomatous‐lymphocytic ILD is a multisystem disease mainly seen in T‐Cell impairment or CVID.^13^, ^27^, ^28^ Bronchiolitis obliterans can occur as a result of CID, PAD, CVID, WAS, DiGeorge syndrome (DGS), and XLP.^13^, ^20^ Fibrosis‐like lung changes and lung emphysema indicate the probability of CHH.^9^ Poststenosis pneumonia originating from bronchial congestion resulting from lymphoid‐tissue enlargement can be a flag of reduced serum Ig G2 level.^27^ Furthermore, pulmonary‐related lymphoid hyperplasia can be seen in activated phosphoinositide 3‐kinase δ syndrome (APDS) and CVID.^6^, ^29^

## 3 EAR, NOSE, THROAT (ENT)

### 3.1 Common

Hearing loss can be a clinical manifestation in patients with Kabuki Syndrome, glucose‐6‐phosphatase enzyme (G6PC3) deficiency, Reticular Dysgenesis, and ADA deficiency.^10^, ^14^, ^30^, ^31^ A diagnosis of sensorineural hearing loss by a specialist might suggest the presence of HIES with *PGM3* mutations and DGS.^32^, ^33^ Chronic otitis media and deafness are the most frequent long‐term complications of PAD.^34^ DGS can predispose the patient to chronic otitis media.^21^ Recurrent otitis media is seen in SIgAD, CVID, XLA, severe combined immunodeficiency (SCID), WAS, defects of complement components (C2, C3, H, I, and P), THI, Ig GSD, PCD, and HIES.^17^, ^20^, ^21^, ^32^ Recurrent otitis externa is manifested in XLA and CGD.^21^, ^35^

Pharyngitis is reported in SCID.^36^ Severe pharyngitis and laryngitis are correlated with the occurrence of HIES and SIgAD.^32^, ^37^, ^38^ Tonsillitis is noted in patients with CVID, APDS, and x‐linked HIGM.^35^, ^39^, ^40^ Rhinitis tonsillitis, adenotonsillitis, and peritonsillar infections should alert physicians to HIES, SIgAD, and hyper IgM syndrome (HIGM), respectively.^17^, ^32^ Patients with SCID also have a high risk of developing rhinitis tonsillitis and adenotonsillitis.^36^ Peritonsillar abscess is reported in CVID.^41^ An association between adenotonsillar hypertrophy resulting in upper airway obstructions and AT, activation‐induced cytidine deaminase (AID) and Uracil‐DNA glycosylase (UNG) deficiency, and also APDS has been reported.^17^, ^21^, ^40^, ^42^ Parotitis is the reason for ENT clinical evaluation in most SIgAD, isolated IgG3 subclass deficiency, and CVID cases.^17^, ^43^

Mastoiditis is reported in SIgAD, WAS, XLA, CVID, CGD, and SCID. Nasal polyposis can guide specialists to consider PCD, TAP deficiency, and CF.^17^, ^44^ Chronic rhinosinusitis is a feature of complement deficiency, XLA, SIgAD, PCD, CF, HIES, DGS, CVID, and SAD.^17^, ^20^, ^21^, ^32^ SCID and AT predispose the patient to recurrent sinusitis.^21^ Allergic rhinitis is reported in SIgMD.^10^ Persistent rhinitis and chronic inflammation of the nares are noted in patients with CGD.^21^, ^35^ Chronic rhinitis is detected in infants with DGS.^35^

### 3.2 Uncommon

PAD can raise suspicion of scarred tympanic membranes.^16^ Hemotympanum is noted in WAS.^35^ Recurrent otorrhea is seen in Ig GSD, specific antibody deficiency (SAD), XLA, or SCID.^17^, ^35^, ^36^, ^37^ Literature also enlists transporter associated with antigen presentation (TAP) deficiency and HIES as causes of nasal septum perforation.^17^, ^44^ Choanal atresia is found in CHARGE (coloboma, heart defect, atresia of the choanae, retardation, genital, and ear abnormalities) syndrome.^14^

## 4 OPHTHALMOLOGICAL

### 4.1 Common

Conjunctivitis can be a potent clue for the diagnosis of PAD.^45^ Conjunctivitis is observed in SIgAD, SIgMD, WAS, autoimmune polyendocrinopathy candidiasis ectodermal dystrophy (APECED), and LAD type I. Bacterial conjunctivitis and keratoconjunctivitis caused by *Hemophilus influenzae* and *Chlamydia trachomatis* are specifically suggestive of XLA. Blepharokerato conjunctivitis is detected in CGD. Corneal scarring, secondary to conjunctivitis, is known as a complication of CGD, WAS, APECED, and XLA. Ophthalmologists who encounter patients suffering from bilateral keratitis should be aware of APECED. Ulcerative keratitis may suggest CGD and WAS.^10^ Macular edema and uveitis are characteristic of CGD. Uveitis is reported in Blau syndrome, CVID, and mevalonate kinase deficiency (MKD). If recurrent hordeola secondary to *Staphylococcus aureus* and blepharitis are detected in a patient, SIgMD is among the first in the differential diagnosis list.^10^

### 4.2 Uncommon

Vitreous hemorrhage is seen in CGD or CVID.^1^, ^10^ Retinal detachment can be indicative of CGD, chronic mucocutaneous candidiasis (CMC), and rarely HIES. Retinitis pigmentosa may precede the diagnosis of CVID and CMC.^10^ Awareness of retinal vasculitis and bilateral optic neuritis may help clinicians to diagnose CVID. Optic atrophy and myopia are mentioned in CMC. Optic nerve pallor and eyelid dermatitis are documented as clinical findings of CGD. Tortuous retinal vessels, tilted optic nerves, eyelid hooding, strabismus, ptosis, and amblyopia are hallmarks of DGS. WAS can exhibit necrotizing eyelid eruptions and eyelid eczema.^10^

## 5 HEMATOLOGIC AND ONCOLOGICAL

### 5.1 Common

Pancytopenia has been reported to develop in patients with CHD and ALPS.^17^, ^18^ Autoimmune cytopenia is seen in APDS, autoimmune lymphoproliferative syndrome (ALPS), CVID, SIgAD, STAT1 GOF, *STAT3* GOF, DGS, immune dysregulation polyendocrinopathy enteropathy X‐linked (IPEX), CTLA‐4 haploinsufficiency and deficiency of LRBA, WAS, dominant‐negative defects in *CARD11*, *ICOS* deficiency, purine nucleoside phosphorylase (PNP) deficiency, and SCID (secondary to hypomorphic mutations such as *RAG1* and *ORAI1* mutations).^6^, ^46^ Auto‐immune hemolytic anemia and immune thrombocytopenia are reported in CVID, SIgAD, and ALPS.^6^, ^16^ Hemolytic anemia is a finding in CHH, IPEX, WAS, and CVID. CVID can also manifest with pernicious anemia.^5^, ^9^, ^47^, ^48^ Aplastic anemia is reported as a rare finding in XLP.^49^ Patients with Shwachman–Diamond syndrome (SDS) can present with macrocytic or normocytic anemia.^50^ Low iron blood levels are assessed in patients with CMC.^17^ Neutropenia can be a clinical feature of CHH, GS2, WAS, DGS, WHIM (warts, hypogammaglobulinemia, infections, and myelokathexis) syndrome, and Cohen Syndrome. Autoimmune neutropenia is detected in X‐linked CD40 ligand deficiency (the most common cause of HIGM) and CVID.^6^, ^9^, ^10^, ^14^, ^33^, ^48^, ^51^ Patients with XLA can be diagnosed with neutropenia, which is responsive to regular intravenous immunoglobulin (IVIG) replacement.^52^ Platelet defects are documented complications of CHD and WAS.^17^, ^53^ IPEX and SDS can manifest thrombocytopenia.^5^, ^50^ Micro thrombocytopenia is seen in WAS.^54^ Idiopathic thrombocytopenic purpura (ITP) is reported in CHH, CVID, and DGS.^9^, ^10^, ^33^ Eosinophilia has associations with Omenn syndrome and HIES.^11^, ^55^

Lymphoma can serve as a diagnostic clue to XLP, AT, CVID, NBS, Bloom syndrome, ALPS, DNA ligase IV deficiency, WAS, and CHH. AD‐HIES with *STAT3* mutations are characterized by a predisposition toward the onset of non‐Hodgkin lymphoma, Burkitt lymphoma, disseminated anaplastic lymphoma, as well as diffuse large B‐cell lymphoma.^9^, ^10^, ^17^, ^18^, ^32^, ^56^ Specialists who encounter patients suffering from mucosa‐associated lymphoid tissue (MALT) lymphoma should be aware of DGS and CVID.^56^ Epstein–Barr (EBV) virus‐associated lymphoma is reported in Artemis syndrome, DGS, RAS guanyl‐releasing protein 1 (RASGRP1) deficiency, CD70 and CD27 deficiencies, XLP, mutations in *SH2D1A*, *XIAP*, and *ITK*, deficiencies of *PRKCD*, *MST1*, *Coronin‐1A, CTPS1, CD137, MAGT1*, or *CARMIL2*.^18^, ^56^, ^57^ Leukemia accounts for the majority of malignancies in patients with WAS, NBS, Bloom syndrome, DNA ligase IV deficiency, and Ig GSD.^10^, ^18^, ^56^ AT can predispose the patient to AML and ALL.^56^ Myelodysplastic syndrome (MDS) and acute lymphocytic leukemia (ALL) are sporadically detected in WAS, whereas *GATA2* deficiency and SDS can exhibit MDS and acute myeloid leukemia (AML).^18^, ^50^, ^56^

Gastrointestinal (GI), skin, breast, lung, and endometrial cancers are suggested to indicate Bloom syndrome. Patients with CVID are also prone to gastric carcinoma. Lynch syndrome (LS), caused by mutations in any 1 of 4 genes (*MLH1*, *MSH2*, *MSH6*, and *PMS2*), can be associated with gastric, colorectal, ovarian, and endometrial cancers.^58^ Patients with CHH or bone marrow failure syndromes have an increased risk of skin cancers. Individuals with AT and bone marrow failure syndromes are more susceptible to female breast cancer.^9^, ^18^, ^47^, ^56^, ^59^ HPV‐related papilloma cancer is seen in WHIM syndrome or *EVER1, EVER2, MST1, RHOH, MAGT1, ITK* deficiencies.^14^ Squamous cell carcinoma (SCC) and BCC is reported in HIES due to *DOCK8* deficiency.^60^, ^61^

### 5.2 Uncommon

Medulloblastoma qualifies patients for further consideration of Bloom syndrome and NBS.^56^ NBS can also evolve into dysgerminoma, glioma, and meningioma, while Bloom syndrome can predispose individuals to osteosarcoma. Neuroblastoma and thyroid cancer should alert physicians to NBS, AT, and DGS.^18^, ^21^, ^56^ Patients with AT have a predisposition to parotid cancer.^21^ Wilms tumor is seen in Bloom syndrome and DGS. Furthermore, clinicians should be alert for DGS when patients have hepatoblastoma, ALL, or teratoid/rhabdoid tumors.^56^ Kaposi sarcoma is seen in OX40 deficiency.^14^

Congenital asplenia is reported in isolated congenital asplenia. Thymoma is described as a feature of Good Syndrome.^14^ Absent or hypoplastic thymus and abnormal thymic epithelium on chest X‐ray or computed tomography could guide physicians to consider SCID, DGS, AT, defects of antigen presentation, *PAX1* deficiency, and Winged helix deficiency.^1^, ^21^, ^62^

## 6 DERMATOLOGIC

### 6.1 Common

Atopic dermatitis (AD) is reported to be correlated with AD‐HIES (job's syndrome), IPEX, Netherton syndrome, PGM3 deficiency, DOCK8 deficiency, *STAT3* GOF, Omenn syndrome, activated leukocyte cell adhesion molecule (ALCAM), and hypereosinophilia.^6^, ^11^, ^63^ The eczematous‐like rash is presented in IPEX, WAS, Omenn syndrome, CHH, or Netherton syndrome.^6^, ^9^, ^16^ Urticaria‐like rashes can appear in patients with APECED, PLAID, *ADGRE2* mutation, familial cold autoinflammatory syndrome (FCAS), Muckle‐Wells syndrome (MWS), and neonatal‐onset multisystem inflammatory syndrome (NOMID).^6^, ^11^ Recurrent urticarial rashes are reported in HIES (Hiob syndrome or Buckley syndrome).^64^ Sun‐sensitive skin lesions and hypo or hyperpigmented skin lesions are detected in Bloom syndrome. Patients with CGD also have an increased susceptibility to photosensitive rashes.^6^, ^18^ Papulo‐erythematous skin rash is the clinical hallmark of Blau syndrome.^10^ Petechiae and purpura serve as a hint to identify WAS.^65^ Telangiectasia is a common feature in AT‐like disordersand Bloom syndrome.^16^, ^18^, ^65^

Poor wound repair is reported in LAD, *Rac2* mutation, and *WDR1* deficiency.^14^, ^66^ Increased susceptibility to severe acne and cellulitis qualifies patients for further consideration of CGD.^17^ Cutaneous viral infections are described in *CARD11*, *LCK*, NEMO, *GATA2, STK4*, *DOCK8*, and *STAT2* deficiencies, *STAT1* GOF, Netherton syndrome, WHIM syndrome, and WILD (warts, depressed cell‐mediated immunity, primary lymphedema, and anogenital dysplasia) syndrome.^6^, ^11^, ^14^, ^67^ Boils/soft tissue abscesses may provide clues to the diagnosis of CGD, HIES, or LAD.^16^

Alopecia is reported in dominant‐negative defects in *CARD11*, STAT1 GOF, CVID, and XLA.^6^, ^51^ Scarring alopecia secondary to hyperkeratotic lesions is seen in CMC.^17^ Diffuse alopecia, along with erythroderma, can be a clinician's guide to the diagnosis of SCID, Omenn syndrome, and hypereosinophilia.^65^ The finding of thickened, fragmented, and discolored nails, with erythema and edema in the surrounding periungual tissue, highlights the need to evaluate CMC. A dermatologist may visit a patient with PLS or dyskeratosis congenita (DKC) due to complaints of nail dystrophy.^10^, ^17^

### 6.2 Uncommon

Cafe.au.lait spots are detected in WAS, NBS, immunodeficiency with centromeric instability and facial anomalies (ICF) syndrome, PMS2, and MSH2 deficiencies.^14^ ADA2 deficiency is characterized by increased susceptibility to livedo reticularis.^68^ Ichthyosis and trichorrhexis invaginatum are seen in Netherton syndrome.^11^

Necrotizing granulomatous skin ulcerations similar to granulomatous polyangiitis can be diagnosed in patients with major histocompatibility complex (MHC) I deficiency. Additionally, inflammatory granulomatous skin lesions are reported in CHH, CVID, HIES, and AT.^6^, ^9^, ^69^, ^70^, ^71^ Erythematous, hyperkeratotic, and serpiginous plaques can be a reason for dermatological evaluation in patients with CMC.^17^ Palmar‐plantar hyperkeratosis is mentioned in Papillon–Lefèvre syndrome (PLS).^10^ Psoriasis plaques are developed in patients with STAT1 GOF and CHH.^6^, ^9^, ^72^ Vitiligo is detected in STAT1 GOF and rarely DGS.^6^, ^33^ Discoid lupus can be developed in female carriers of X‐linked CGD and complement deficiencies.^6^, ^73^ Scleroderma may be seen in *STAT3* GOF.^6^

## 7 GI

### 7.1 Common

Gastroesophageal reflux is mentioned in DGS, Bloom syndrome, and *STAT3*‐HIES. Esophageal dysmotility and severe dysphagia are characteristic of *STAT3*‐HIES and DGS, respectively.^18^, ^33^, ^74^ Gastric diseases, including corpus‐predominant autoimmune gastritis, autoimmune pan‐gastritis, and lymphocytic gastritis, are detected in CVID and APECED.^5^

The liver undergoes many changes in CVID, including abnormal liver biochemistries, primarily increased levels of alkaline phosphatase (ALP), nodular regenerative hyperplasia (NRH), and liver cirrhosis.^75^ XLA and APDS can also predispose the patient to NRH.^76^, ^77^ A diagnosis of sclerosing cholangitis and cirrhosis by a gastroenterologist may suggest the presence of CD40 ligand deficiency.^1^ Hepatitis is reported in APDS, APECED, IPEX, X‐linked HIGM, STAT1 GOF, and *STAT3* GOF.^6^, ^16^ CHH and CGD can exhibit hepatosplenomegaly. Moreover, splenomegaly is detected in CVID, ALPS, hypereosinophilia, and CHH.^6^, ^9^, ^16^, ^35^, ^65^

Constipation is a common complication among patients with DGS.^33^ Diarrhea accompanied by constipation, abdominal pain, and failure to thrive can be developed in 32%–48% of patients with CGD. Recurrent diarrhea is a characteristic of XLA, SCID, defects of antigen presentation, and CHH.^5^, ^9^, ^21^ Watery diarrhea is seen in CVID.^5^ Patients with SDS can present with steatorrhea.^50^ Inflammatory bowel disease (IBD) is a clinical diagnosis that often brings patients with WAS, CVID, X‐linked inhibitor of apoptosis (XIAP) deficiency, NEMO deficiency, CGD, or XLA to medical centers.^6^, ^26^, ^78^ Colitis is a clinical finding in CVID, dominant‐negative defects in *CARD11*, and CGD.^6^ CVID and SIgAD could simulate celiac disease. CVID can also cause patchy chronic active enterocolitis accompanied by lymphoid hyperplasia and granulomata, which resemble Crohn's disease.^5^ G. lamblia is recognized as the leading cause of gastroenteritis in CVID.^79^ Giardiasis and strongyloidiasis are detected in SIgAD. Patients with XLA may also suffer from chronic giardiasis.^5^

### 7.2 Uncommon

DGS can rarely develop laryngeal web, esophageal atresia, and tracheoesophageal fistula.^33^ The pyogenic liver abscess is diagnosed in PLS and CGD.^10^, ^80^ Veno‐occlusive disease with immunodeficiency (VODI) syndrome can be associated with hepatomegaly and veno‐occlusive disease.^14^ Investigation of exocrine pancreatic insufficiency and malabsorption may provide a guide to diagnosing SDS.^1^ GI‐related lymphoid hyperplasia is found in APDS.^6^ Autoimmune enteropathy is seen in CHH, CTLA‐4 haploinsufficiency and deficiency of LRBA, IPEX, and *STAT3* GOF.^5^, ^9^ *STAT3*‐HIES has an increased susceptibility to spontaneous intestinal perforations and less likely fungal infections of the GI tract (typically *histoplasmosis*, *Cryptococcus*, and *Coccidioides*).^74^ Intestinal dysfunction is detected in APECED.^6^ Intestinal malrotation, Hirschsprung disease, imperforate anus, and the umbilical, inguinal, and diaphragmatic hernia may serve as a hint to identify DGS.^33^ Intestinal obstruction and vomiting are identified in immunodeficiency with multiple intestinal atresia.^81^ Early‐onset enteric fistula is reported in IL10, IL10RA, and IL10RB deficiencies.^14^

## 8 ENDOCRINE

Hashimoto's thyroiditis is diagnosed in patients with IPEX. Autoimmune thyroiditis is associated with milder forms of ADA deficiency. In general, thyroid disease should be taken into consideration in patients with STAT1 GOF and Schimke immuno‐osseous dysplasia (SIOD).^6^, ^10^, ^82^ Hypo‐ or hyperthyroidism is investigated in CHH and rarely DGS.^9^, ^33^ SIgAD, IPEX, and WAS are the three main thyroiditis‐associated IEI.^4^ Hypoparathyroidism and consequent hypocalcemia are the most common endocrinopathy in DGS, chromosome 10p13‐p14 deletion syndrome (10p13‐p14DS), APECED, CMC, CHH, and CVID.^4^, ^9^, ^21^, ^83^ Osteoporosis compromises the quality of life in patients with CVID.^47^, ^84^

The risk of type 1 diabetes (T1D) is higher in patients with IPEX (FOXP3, forkhead box P3), WHIM syndrome, autoimmune polyglandular syndrome type 1 (APS type 1 due to AIRE mutation), CTLA4 mutation, deficiency of LRBA, milder forms of ADA deficiency, and STAT1 GOF. It should not be overlooked to assess intensive genetic analysis, including IEI‐related genes in a patient whose onset of T1D is before six months of age.^4^, ^6^ Type 2 diabetes (T2D) development is noticed in AT.^82^ Generally, AT, CF, IEI with DNA repair impairments, and Bloom syndrome can have an association with diabetes mellitus.^4^, ^85^

Adrenal insufficiency due to the autoimmune inflammation is probable in the nuclear factor kappa B (NF‐κB) transcription factors dysfunction, mutations in *MCM4*, AIRE, or CTLA4, and DAVID syndrome (CVID accompanied by adrenocorticotropic hormone (ACTH) insufficiency). Meanwhile, adrenal insufficiency due to adrenal hypoplasia is associated with MIRAGE (myelodysplasia, infection, restriction of growth, adrenal hypoplasia, genital phenotypes, and enteropathy) syndrome due to SAMD9/SAMD9L gene mutations.^4^

## 9 REPRODUCTIVE SYSTEM AND THE UROLOGICAL

An absent uterus is investigated in DGS.^33^ Premature ovarian failure develops in APECED and CVID.^4^, ^86^ A diagnosis of ovarian insufficiency with severe neutropenia may indicate Kostmann disease due to HAX1 gene mutations.^4^ Pubertal delay is seen in AT.^18^ Early menopause is reported in Bloom syndrome.^18^ Bloom syndrome, CF, and CHH can predispose the patient to infertility.^14^, ^85^ Micropenis and the absence of pubertal development are observed in CHARGE syndrome (Heterozygous CHD7 gene deficiency).^4^ Cryptorchidism, together with hypospadias, is reported in DGS. Individuals with Kabuki Syndrome could develop urinary tract malformation and premature thelarche.^33^ Urogenital malformation is also reported in GATA2 deficiency.^87^

## 10 RHEUMATOLOGIC

Arthritis is exhibited in X‐linked CD40L deficiency, SIgAD, *STAT3* GOF, Blau Syndrome, APDS, MHC I deficiency, ALPS, DGS, IPEX, WAS, XLA, *ICOS* deficiency, PNP deficiency, and SCID.^6^, ^16^ Juvenile idiopathic arthritis (JIA) is noted in CHH and CVID.^9^, ^10^, ^88^ Septic arthritis is reported in XLA.^21^ Vasculitis is seen in MHC I deficiency, WAS, Blau syndrome, IPEX, HIES with DOCK8‐deficiency, and rarely CVID.^6^, ^10^, ^32^

Systemic lupus erythematosus is detected in SIgAD, deficiencies in the early components of classical complement pathways (C1, C2, and C4), STAT1 GOF, and CVID.^6^ Oral ulcers occur in patients with CGD, CVID, SCID, and HIGM. Aphthous ulcers can be developed in X‐linked CD40L deficiency.^6^, ^17^, ^21^ The Sjogren's‐like disease is reported in APECED and CVID.^6^, ^88^ Piezogenic pedal papules and joint hyperextensibility are noticed in *GATA2* deficiency.^89^

## 11 RENAL

Glomerulonephritis is observed in APDS.^6^ Lupus‐like glomerulonephritis can be formed in deficiency of early components of the classical complement pathway.^16^ Membranoproliferative glomerulonephritis type 2 (MPGN2) and atypical hemolytic‐uremic syndrome (HUS) should raise suspicion toward complement factor H deficiency.^10^ IgA nephropathy is seen in WAS.^90^ Progressive renal failure can occur in SIOD.^10^ Renal anomalies/agenesis and hydronephrosis are identified in DGS.^33^ Granuloma of the kidneys is reported in Blau syndrome.^10^ Vesico‐renal‐genital anomaly is manifested in CHARGE syndrome, G6PC3 deficiency, and Malpuech, Michels, Mingarelli, and Carnevale (3MC) syndrome.^14^

## 12 CARDIAC

Interrupted aortic arch is noticed in DGS.^21^ Aneurysms are indicated in all subtypes of HIES and *STAT1* GOF (due to heterozygous variants in the STAT1 gene).^6^, ^32^ Pseudoaneurysms, congenital patent ductus venosus, superior vena cava syndrome, vasculitides, vascular ectasia, and thrombosis are noted in all subtypes of HIES.^32^ Patients with *STAT3*‐mutated HIES may be visited by a cardiologist due to coronary artery tortuosity or dilation.^32^ Venous angeictasis is revealed in G6PC3 deficiency.^14^ Angioedema is reported in C1 inhibitor and factor XII deficiencies.^14^

The finding of peak systolic aortic pressure in high cutoff levels and mild valvular regurgitation in patients with CVID highlights the need for regular cardiac monitoring to control the sequels such as pulmonary arterial hypertension.^91^ Murmur or cyanosis suggestive of congenital heart defects (particularly conotruncal anomalies) is associated with DGS, severe congenital neutropenia (SCN) type 4, CHARGE syndrome, Kabuki Syndrome, and *MST1/STK4* deficiency.^1^, ^10^, ^16^, ^33^ G6PC3 deficiency could be characterized by a congenital heart disorder.^14^ Pericarditis is a cardiac finding in patients with APDS.^6^

IFNAR1 deficiency could predispose the patient to multisystem inflammatory syndrome (MISC) after coronavirus disease (COVID‐19).^92^

## 13 FACIAL

Individuals with DGS are at high risk of developing palatal abnormalities like velopharyngeal incompetence, overt cleft palate, cleft lip with or without cleft palate, and Pierre Robin, as well as subtle dysmorphic features.^33^ A distinctive facial appearance along with a high and prominent forehead, prognathism or retrognathism, enlargement of the interalar distance, thickening of the soft tissues of the ear or nose, and the high arched palate can be suggestive of HIES.^32^, ^93^ A rough skin appearance with exaggerated pore size is mentioned in HIES with *STAT3* mutation. This characteristic appearance becomes evident with age.^94^ It is suggested that suspicions of HIES with *PGM3* mutations should increase when wide nostrils and prominent lips develop.^32^ NBS is characterized by a bird‐like face.^10^

In conclusion, if facial abnormalities are observed in a neonate, DGS, ICF syndrome, 3MC syndrome type 2 (*COLEC11* deficiency), Cohen syndrome (*COH1* deficiency), *ITCH* deficiency, *STAT5B* deficiency, FILS (facial dysmorphism, immunodeficiency, livedo, and short stature) syndrome, *RNF168* deficiency, Kabuki Syndrome, Cernunnos (XLF), and Bloom syndrome are among the first in the differential diagnosis list.^1^, ^10^, ^95^

## 14 ODONATOLOGICAL

Retention of deciduous teeth is one of the clinical findings in HIES. A delay in the shedding of primary teeth is seen in *STAT3* deficiency.^1^, ^93^ Patients’ dental enamel hypoplasia may suggest an onset of APECED or DGS.^1^, ^33^ Gingivitis is manifested in neutrophil deficiency or CGD.^16^, ^17^ Intermittent stomatitis is reported in HIGM and cyclic neutropenia.^17^, ^35^, ^96^ Ulcerative stomatitis is noted in CGD.^35^ Severe periodontitis and, consequently, premature loss of teeth are reported in CHD, LAD type I, and PLS.^10^, ^17^

## 15 PSYCHIATRIC AND INTELLECTUAL CHARACTERISTICS

Several neurocognitive disorders among patients with DGS are reported, including motor delays (often with hypotonia) and speech/language deficits (in infancy), and learning difficulties (in childhood).^33^ Cognitive impairment coinciding with a neurological defect and a myelodysplastic syndrome should alert physicians to Kostmann disease (*HAX1* gene mutation).^1^ AT and syndromic CIDs can predispose individuals to cognitive defects.^14^, ^55^ Developmental delay is indicated in 3MC syndrome, LAD type 2, Cohen syndrome, Kabuki syndrome, Barth syndrome, P14 deficiency, adenosine deaminase acting on RNA 1 (ADAR1) deficiency, HIES with *PGM3* mutations, PNP deficiency, MKD, DGS, NBS, and *ITCH* deficiency.^1^, ^10^, ^32^ Mild to moderate intellectual disability is seen in DGS, b‐actin deficiency, HIES with *PGM3* mutations, Kabuki Syndrome, Bloom syndrome, NBS, CHD, and ICF syndrome.^1^, ^10^, ^17^, ^18^, ^32^, ^33^ A variety of psychiatric disorders like attention deficit with hyperactivity disorder (ADHD), autism spectrum disorder (ASD), anxiety, mood disorders, and schizophrenia can be associated with DGS.^33^

## 16 NEUROLOGICAL

### 16.1 Common

Meningitis is reported in PAD, XLA, WAS, HIES with DOCK8‐deficiency, and complement deficiency.^21^, ^32^, ^45^, ^97^ Chronic enteroviral meningoencephalitis is revealed in XLA.^45^ Encephalopathy and encephalomyelitis are seen in CVID.^98^ Viral encephalitis could be a clinical feature of *TLR3, UNC93B1, TRAF3, TRIF, TBK1* deficiencies.^14^ Hypo‐calcemic seizures are one of the presentations of DGS.^1^ Seizure is seen in Kabuki syndrome.^10^

An association between peripheral neuropathy or myelopathy and CVID has been documented previously.^98^ Peripheral neuropathy is also reported in CHD.^17^ Cranial neuropathy is detected in Blau Syndrome.^10^ Multifocal motor axonal neuropathy and narcolepsy guide neurologists to consider CHH.^9^ Fibromyalgia is noted in IgGSD.^99^ Aneurysms often found incidentally in brain imaging are highly suggestive of *STAT3*‐mutated HIES.^1^ Brain infarction is indicative for HIES with DOCK8‐deficiency.^32^ Patients with ADA2 deficiency can manifest increased susceptibility to CNS involvement in the form of ischemic or hemorrhagic strokes.^68^ Central nervous system (CNS) vasculitis is noticed in XLP.^49^ Spontaneous CNS candidiasis is reported in *CARD9* deficiency.^100^

### 16.2 Uncommon

Intracranial calcifications, cerebral atrophy, and leukodystrophy should alert physicians to spondylo‐enchondro‐dysplasia with immune dysregulation, ADAR1 deficiency, *SAMHD1* deficiency, and type 1 interferonopathies.^1^

After polyomavirus JC infection, pyramidal signs can reveal progressive multifocal leukoencephalopathy suggestive of PNP deficiency.^1^ Ataxic gate and an increased serum level of a‐fetoprotein are seen in AT, AT‐like disease, PNP deficiency, and *RNF168* deficiency.^1^ Ataxia and hypotonia, dysarthria, demyelination‐associated neurocognitive impairment, and myoclonus are mentioned in HIES with *PGM3* mutations.^11^, ^32^ Progressive cerebellar ataxia is investigated in MKD.^10^ Microcephaly is reported in NBS, DGS, *DKC1*, *RNF168*, RAD50, DNA ligase IV, Cernunnos (XLF), and DNA‐dependent protein kinase catalytic subunit (DNAPKcs) deficiencies.^1^, ^16^, ^33^

## 17 ORTHOPEDIC

### 17.1 Common

Fractures following minor trauma, scoliosis, osteoporosis, degenerative spine disease, craniosynostosis, or hyper‐extensive joints can suggest a HIES diagnosis. Scoliosis, craniosynostosis, and cervical and thoracic vertebral anomalies are seen in DGS.^1^, ^32^, ^33^, ^93^ Scoliosis is demonstrated in HIES with *PGM3* mutations and Kabuki syndrome.^10^, ^32^ Bone and joint infections such as Mycoplasma arthritis are detected in CVID or XLA.^45^ Osteomyelitis is noted in CGD and XLA.^35^ DIRA (deficiency of the interleukin 1 receptor antagonist) syndrome secondary to defects in *IL1RN* might develop neonatal sterile multifocal osteomyelitis.^1^ Severe fungal osteomyelitis of the skull base is a rare manifestation of myeloperoxidase (MPO) deficiency.^17^

### 17.2 Uncommon

Dystrophy is reported in the CANDLE (Chronic Atypical Neutrophilic Dermatosis with Lipodystrophy and Elevated temperature) syndrome. Chondrodysplasia and short stature might occur in SDS, while skeletal dysplasia, short stature, and limb dwarfism are mentioned in *RNF168, MCM4, ACTB, ACP5, STAT5B, FILS, SMARCAL1, RMPR*, and *BLM* gene mutations.^1^, ^50^ Costochondral junction flaring is identified in ADA deficiency.^1^ If the physician encounters a patient with spondylo‐epiphyseal dysplasia, then SIOD should be ruled out.^10^ Finger and spinal deformities, slipped capital femoral epiphysis, and rib cage abnormalities are reported in SDS.^50^ Hip dislocation is a clinical feature of Kabuki syndrome.^10^ Camptodactyly is illustrated in Blau syndrome, whereas DGS can rarely develop clubfoot and polydactyly.^10^, ^33^

## 18 OTHER

Disseminated lymphadenopathy is explored in CVID, Omenn syndrome, ALPS, or autosomal recessive HIGM.^16^ Nonmalignant lymphadenopathy is marked in ALPS.^6^ Cervical lymphadenopathy is noted in WAS, interferon‐gamma (IFN‐gamma)/interleukin‐12 (IL‐12) pathway defects, and CGD.^21^, ^35^ Lymphadenitis is described in HIES and cyclic neutropenia.^35^, ^64^ Absent lymph nodes are detected in XLA or SCID. XLA cases are at high risk of developing the absence of tonsils.^16^ The mycobacterial abscess is reported in IFN‐gamma/IL‐12 pathway defects.^21^ Disseminated infectious mononucleosis is reported in XLP.^21^ Vaccine‐associated poliomyelitis can occur in XLA.^21^ CVID and SCID are associated with paralysis or diarrhea after taking the poliovirus vaccine.^14^ Suspicions of CMC should increase when white adherent plaques of thrush or angular cheilitis (or perlèche) are detected.^17^ Leukoplakia of the oral mucosa is manifested in DKC.^10^ Epistaxis and oral bleeding are seen in WAS.^65^ Patients with WAS show a predisposition to prolonged bleeding after circumcision.^21^ X‐linked DKC, Schimke syndrome, and syndromic diarrhea/tricho‐hepato‐enteric syndrome (SD/THE) are characterized by Intrauterine growth retardation.^14^, ^101^ Intrauterine polyhydramnios is reported in immunodeficiency with multiple intestinal atresia.^14^ THI can be associated with prematurely born.^21^ Delayed separation of the umbilical cord is the clinical hallmark of LADs and *Rac2* deficiency.^14^ Coarse face is presented in autosomal dominant HIES.^14^ NEMO, *STIM1*, and *ORAI1* deficiencies could be characterized by anhidrotic ectodermal dysplasia.^14^ Hyponatremic dehydration is a clinical feature of CF.^85^ Hypertension due to atypical hemolytic uremic is diagnosed in CD46, factor B, factor I, factor H, factor H‐related protein, and thrombomodulin deficiencies.^14^ High fever indicates the probability of NOMID. Recurrent febrile attacks should alert physicians toward MKD and familial Mediterranean fever.^10^, ^102^ Associated sarcoid‐like granulomatous disease is detected in AT, SCID, and CVID.^103^, ^104^ Secondary amyloidosis is manifested in NOMID.^10^

## 19 CONCLUSION

Recently, many advances have emerged in the realms of both diagnosis and treatment aspects of IEI.^2^ Since heterogeneous presentations of IEI pose diagnostic challenges for physicians, shedding light on the organ's involvement in IEI is an ever‐important issue. In this review, we studied the manifestations of IEI in each part of the body as a whole to provide meaningful insights regarding the physician's clinical decision. Sometimes, characteristic features of organs such as the ear, blood, or skin could prompt the physician to consider IEI among the first in the differential diagnosis list. Meanwhile, due to the potential involvement of the lung, GI tract, and endocrine glands, awareness of their associated symptoms has contributed to greater comprehension of the following sequels. The current review shows that several manifestations of IEI can be misinterpreted as separate diseases and cause mismanagement.

Based on the previously published literature, many presentations are not routinely expected in patients with IEI. Accordingly, articles and tables containing all the symptoms reported in various types of IEI can provide a practical guide to the treating physician and specialists to define a proper diagnostic strategy and therapeutic protocols. The national and continent‐based cohorts and different geographical reports of diagnostic signs and symptoms in various clinical or genetic forms of IEI are required for further advances. However, it is essential to mention that improved registration efforts and maintenance of the available databases are necessary to conduct future studies.

Regarding the increasing knowledge of IEI‐associated disorders, the latter concern applies to developing targeted diagnostic guidelines for clinicians based on their specialty. It is to be hoped that further elucidation of the related manifestations will ere long have implications for increasing the life expectancy of the patients suffering from IEI.

## AUTHOR CONTRIBUTIONS

**Fatemeh Mohammadi**: Conceptualization; investigation; resources; visualization; writing—original draft. **Amirhossein Yadegar**: Investigation; resources; writing—original draft. **Mahta Mardani**: Resources; writing—original draft. **Aryan Ayati**: resources; writing—original draft. **Hassan Abolhassani**: writing—original draft. **Nima Rezaei**: Conceptualization; supervision; validation; visualization; writing—review and editing.

## CONFLICT OF INTEREST STATEMENT

The authors declare no conflict of interest.

## Data Availability

Data are available on request from the corresponding author.
